# Low-affinity CD4+ T cells are major responders in the primary immune response

**DOI:** 10.1038/ncomms13848

**Published:** 2016-12-15

**Authors:** Ryan J. Martinez, Rakieb Andargachew, Hunter A. Martinez, Brian D. Evavold

**Affiliations:** 1 Department of Microbiology and Immunology, Emory University, 1510 Clifton Rd NE, Atlanta Georgia, 30322, USA

## Abstract

A robust primary immune response has been correlated with the precursor number of antigen-specific T cells, as identified using peptide MHCII tetramers. However, these tetramers identify only the highest-affinity T cells. Here we show the entire CD4+ T-cell repertoire, inclusive of low-affinity T cells missed by tetramers, using a T-cell receptor (TCR) signalling reporter and micropipette assay to quantify naive precursors and expanded populations. *In vivo* limiting dilution assays reveal hundreds more precursor T cells than previously thought, with higher-affinity tetramer-positive T cells, comprising only 5–30% of the total antigen-specific naive repertoire. Lower-affinity T cells maintain their predominance as the primary immune response progresses, with no enhancement of survival of T cells with high-affinity TCRs. These findings demonstrate that affinity for antigen does not control CD4+ T-cell entry into the primary immune response, as a diverse range in affinity is maintained from precursor through peak of T-cell expansion.

## Abstract (web summary)

MHC II tetramers are used to study T cell expansion but they preferentially bind high affinity T cell receptors. Here the authors show that T cells with low affinity receptors expand in number along with the T-cell repertoire and might therefore contribute more to the primary immune response than was thought.

The number of antigen-specific CD4+ T cells in the naive mouse correlates with the effector potential of the population. Defining the total number of antigen-specific T cells in an organism, therefore has important ramifications for understanding immune response outcomes^1^,^2^,^3^,^4^,^5^,^6^. Currently, peptide-major histocompatibility complex (pMHC) tetramers (Tet) provide the gold standard for the identification of antigen-specific CD4+ T cells^7^,^8^. Tetramers are limited to identifying CD4+ T cells with higher-affinity T-cell receptor (TCR):pMHC interactions^9^,^10^,^11^,^12^ and bind via an avidity-dependent mechanism without dependence on CD4 co-receptor^11^,^13^,^14^,^15^,^16^,^17^,^18^. Thus, unbiased assessment of the total number of antigen-specific T cells has been challenging in the case of CD4+ T cells, owing to the high-affinity predisposition by tetramers. Therefore, the contribution of lower-affinity T cells in the naive and expanded T-cell repertoires is currently unknown, in part due to the difficulty of accurately quantifying these T cells in the naive repertoire.

Previous studies have suggested T cells with higher-affinity TCR:pMHC interactions possess enhanced survival or preferred selection during the primary or secondary immune response^19^,^20^,^21^, with others reporting affinity independence of T-cell maintenance during an immune response^22^. These experiments only analysed biased populations by restricting αβ TCR diversity and/or sampling with pMHC tetramers, thereby potentially missing clones participating in the response. Further works using TCR-transgenic (Tg) models and altered peptide ligands support the concept that optimal responses occur in the case of highest-affinity interactions^23^,^24^. Yet, none of these analyses encompass the full polyclonal repertoire, leaving the question on the contribution of lower-affinity and higher-affinity T cells in the expanded T-cell population unanswered.

To study the contribution of low-affinity and high-affinity CD4+ T cells to the primary immune response, the number of naive and expanded total T cells must be identified. Multiple groups have acknowleged the presence of lower-affinity (Tet-negative, Tet−) T cells, but these cells are difficult to adequately quantitate at any point during the immune response^9^,^11^,^25^. To accomplish this task, we repurposed the Nur77^gfp^ TCR signalling reporter as a method for identifying lower-affinity, Tet− antigen-specific CD4+ T cells. To define the number of precursor T cells, we used the Nur77^gfp^ reporter in an *in vivo* limiting dilution assay (LDA), finding Tet− CD4+ T cells made up the majority of the naive antigen-specific T-cell population. On expansion, the ratio of high-affinity to low-affinity antigen-specific CD4+ T cells was reduced, signifying high-affinity TCRs do not confer a clonal expansion advantage. As well, total naive precursor numbers positively correlate with expanded CD4+ T cells, indicating total precursor number predicts expansion when the entire range of TCR affinity is analysed. These data demonstrate T-cell responses are population based with a range of naive affinities that are maintained throughout an immune response to preserve affinity and diversity.

## Results

### LDA reveals similar numbers of Tet− and Tet+ CD4+ T cells

**Figure 1:**
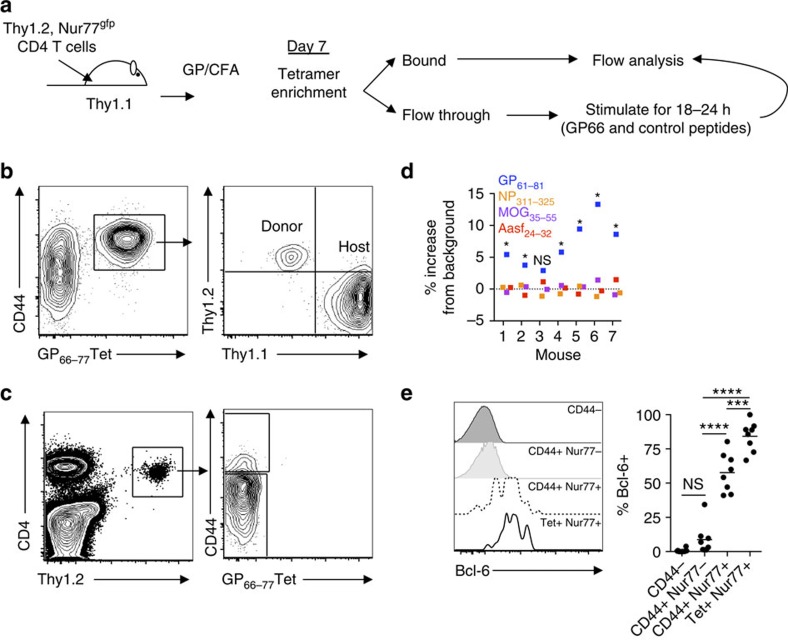
Tet+ LDA reveals low-affinity CD4+ T cells in the naive repertoire. (**a**) 6 × 10^6^ Nur77^gfp^ CD4+ T cells (6 × 10^5^ after a 10% park rate, Thy1.2+) were transferred into congenically distinct (Thy1.1+) mice and immunized with GP_61–81_ peptide/CFA followed by tetramer enrichment and restimulations. (**b**) Flow cytometry of representative GP66-Tet+ enrichment of secondary lymphoid organs from day 7 immunized mice with a positive donor (Thy1.2+) clone identified. (**c**) After tetramer enrichment, the unbound cells were independently stimulated with 100 μg ml^−1^ of specific (GP_61–81_) or non-specific (Aasf_24–32_, MOG_35–55_ or NP_311–325_) peptides and gated to identify donor CD44+ Tet− Thy1.2+ CD4+ T cells by flow cytometry. (**d**) Percent change over averaged background of Nur77^gfp+^CD69+ CD4+ T cells after stimulation with individual antigens (*n*=7, two independent experiments, *=3 s.d. above background average, NS=no significance). (**e**) Bcl-6 expression measured by flow cytometry of CD4+ T cells from day 5 immunized mice within identified subsets. Frequency of cells expressing Bcl-6 within each subset identified (points represent individual mice, *n*=6–7, two experiments, one-way analysis of variance, NS=no significance, ***P<0.001,****P<0.0001).

The transfer of bulk CD4+ T cells at the tetramer-positive (Tet+) limiting dilution level has proven fruitful in the study of single-cell expansion and differentiation^26^,^27^. However, polyclonal antigen-specific CD4+ T cells with lower-affinity TCR:pMHCII interactions are not detected by traditional pMHCII tetramer staining used in these assays^9^,^10^,^28^. Consequently, lower-affinity, antigen-specific CD4+ T cells are missed in these single-clonotype pMHCII tetramer-based analyses. To better define the response inclusive of lower-affinity T cells, the TCR-specific signalling reporter Nur77 was used as a readout of antigen specificity^29^,^30^,^31^. To determine the extent that lower-affinity T cells participate in an immune response, we transferred T cells from Nur77^gfp^ mice at the levels reported to be limiting for Tet+ LCMV GP_66–77_-specific CD4+ T cells (6 × 10^6^ CD4+ Thy1.2+ T cells into congenically distinct Thy1.1+ recipients)^26^. At day 7 post immune challenge with peptide antigen in Complete Freund's adjuvant (CFA) (GP66/CFA; Fig. 1a), GP66-Tet+ CD4+ T cells were enriched and designated as donor (Thy1.2+) or host (Thy1.1+) derived based on their respective Thy1 expression (Fig. 1b, gating strategy Supplementary Fig. 1A). At this number of transferred T cells, four of the seven mice possessed a GP66-Tet+ donor clone, in close agreement with published results^26^. To identify if these mice also contained lower-affinity Tet− cells, the samples were depleted of GP66-Tet+ T cells by tetramer pulldown, and the remaining T cells (Fig. 1c) were stimulated in vitro for 18–24 h with specific (GP_61–81_) or non-specific peptide antigen (Aasf_24–32_, MOG_35–55_, NP_311–325_; Fig. 1a). To assess antigen specificity, the nuclear receptor Nur77 was used as its expression has been shown to be TCR signalling strength dependent^29^. On the basis of Nur77 expression, six of the seven-transferred Tet− CD4+ T-cell populations stimulated with GP66 demonstrated Nur77/CD69 expression with a greater than three s.d. increase above the mean of the non-specific controls (Fig. 1d). There was a low-level background of Nur77 expression that was priming antigen independent (Fig. 1d), but the normalized per cent increase of Nur77/CD69 expression for the GP66-stimulated samples caused the greatest increase. These findings show lower-affinity, Tet− populations are present at least as frequent as Tet+ cells, as demonstrated by similar number of mice with antigen-specific populations (4/7 mice for Tet+, 6/7 mice for Tet−).

Nur77 expression has been used to readout functionality of CD4+ T cells in multiple systems^29^,^30^,^31^,^32^,^33^,^34^,^35^. Even though these T cells are functional, it does not describe the role these T cells are playing in the immune response. Therefore, we interrogated the expression of Bcl-6, the lineage-defining transcription factor for follicular helper T cells (T_FH_), in the Tet+ and Tet− antigen-specific T cells. Bcl-6 expression has been reported to be induced with a variety a range of affinity, with the highest-affinity and lowest-affinity interactions, inducing Bcl-6/T_FH_ development^36^. Antigen-specific T cells showed expression of Bcl-6, regardless of whether they were Tet+ or Tet−, although a greater frequency of higher-affinity, Tet+ T cells expressed Bcl-6 compared with lower-affinity, Tet− antigen-specific T cells (Fig. 1e). Antigen-inexperienced (CD44−) T cells and antigen-experienced but not antigen-specific (CD44+Nur77−) T cells demonstrated little to no expression of Bcl-6 (Fig. 1e). Our data support the findings that T_FH_ differentiation can occur for TCRs with a range of affinities^36^,^37^. As well, the data show that high-affinity and low-affinity T cells have shared, but distinct functions in the total T-cell population.

### Low-affinity T cells predominate the naive repertoire

**Figure 2:**
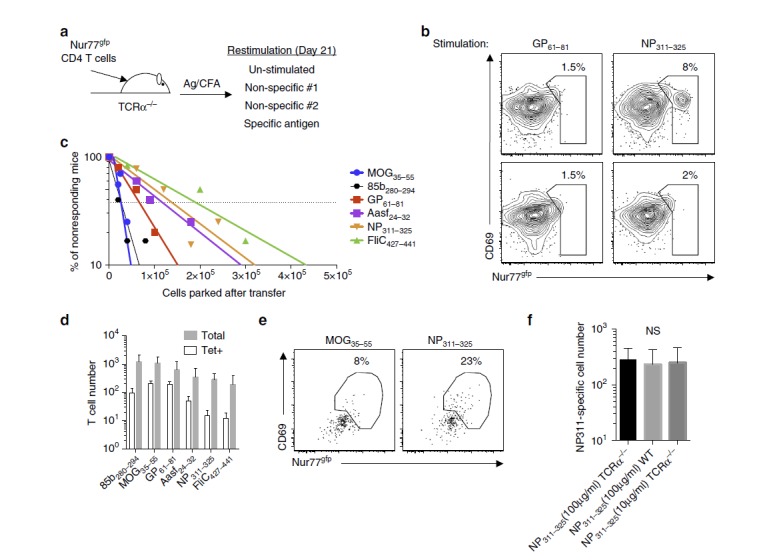
Increased number of naive precursor identified using an *in vivo* LDA. (**a**) Varied numbers of purified Nur77^gfp^ CD4+ T cells were transferred into individual TCRα^−/−^ mice, immunized with peptide antigen in CFA and allowed to expand for 21 days before peptide rechallenge. (**b**) Day 21 flow cytometry analysis of antigen specificity on donor CD4+ T cells after NP_311–325_ immunization showing CD69/Nur77 expression after antigen rechallenge with either control (GP_61–81_-left panel) or priming antigen (NP_311–325_-right panel). NP_311–325_-positive clone shown on top row with negative clone shown on bottom row. (**c**) Positive clones were identified when the antigen-specific upregulation of CD69/Nur77 were three s.d.'s above the background mean and were graphed as a function of initial cells transferred after accounting for a 20% park rate in lymphophenic mice (*n*=4–12 mice per concentration per antigen). Dotted line represents 37% mark of nonresponding mice. (**d**) Tabulated pMHCII tetramer and Nur77 LDA calculated naive CD4+ T cells number for a single antigen, represented as mean±95% confidence interval. FliC and Aasf Tet+ values are taken from previously published work (Marc Jenkins, personal communication, Nelson *et al*.^8^). (**e**) Limiting dilution experiments in WT mice after immunization with NP_311–325_/CFA, with flow cytometry plots showing CD69 and Nur77 expression for background (MOG_35–55_) or antigen-specific stimulation (NP_311–325_; representative sample). (**f**) Enumeration of naive LDA for NP_311–325_-specific T cells in TCRα^−/−^ or WT mice restimulated with 100 μg ml^−1^ of peptide or TCRα^−/−^ mice restimulated with 10 μg ml^−1^ peptide (five independent experiments, three to four T-cell dilutions per experiment, data represented as mean±95% confidence level, one-way analysis of variance, NS=no significance).

Although the above data identified Tet− CD4+ T cells during an immune response, it did not quantitate the initial precursor number of these lower-affinity T cells. We next set out to enumerate the precursor frequency of naive antigen-specific CD4+ T cells independent of pMHCII tetramer by using the Nur77 reporter in an *in vivo* LDA^38^. Varying numbers of CD4+ T cells from Nur77^gfp^ mice were transferred into T-cell-deficient TCRα^−/−^ mice and recipients were immunized with peptides emulsified in CFA (Fig. 2a). Lymphopenic hosts were used as this allowed for larger blast sizes for individual T-cell clones, thereby increasing the sensitivity of the assay as it is dependent on population increases in expression of Nur77. At 21 days post immunization, splenocytes from recipient mice were restimulated *ex vivo* for 18–24 h with specific or non-specific peptide antigens before assessment for Nur77^gfp^ and CD69 expression (Fig. 2b). Representative flow plots of transferred CD4+ T cells that demonstrated positive responses (top row, Fig. 2b) and negative responses (bottom row, Fig. 2b) are shown for NP_311–325_-primed mice. NP_311–325_-stimulated samples containing a Nur77^gfp+^CD69^+^ population greater than three s.d.'s above the mean of two non-specific peptides (GP_61–81_ non-specific peptide control shown) were tabulated as positive and graphed as a function of the number of CD4+ T cells present in the hosts after transfer (Fig. 2c). The points at which 37% of the hosts do not possess a clone equates to where a single precursor cells is present in the population (dotted line, Fig. 2c) and is based on a 20% park rate into lymphopenic mice with a total of 4 × 10^7^ CD4+ T cells per mouse. The precursor frequencies were calculated for six different epitopes (MOG_35–55_ self-antigen: 1,099 (669–1805) cells, MTB 85b_280–294_: 1,206 (682–2,133) cells, LCMV GP_61–81_: 627 (322–1,218) cells, Chlamydia Aasf_24–32_: 350 (169–725) cells, Influenza NP_311–325_: 285 (179–454) cells, and Salmonella FliC_427–441_: 192 (92–402) cells) that were chosen as they spanned the range of previously published tetramer precursor frequencies and were plotted with their 95% confidence levels (Fig. 2d)^8^. Comparison of the LDA and naive tetramer enrichments revealed Tet+ numbers accounted for 5–30% of the total naive antigen-specific repertoire, demonstrating tetramer only identifies a minor subset of each antigen-specific T cells in a naive population. Control LDA experiments were performed for NP_311–325_ antigen in wild-type (WT) mice and in TCRα^−/−^ mice, when using only 10 μg ml^−1^ of peptide for restimulation, instead of 100 μg ml^−1^ as for previous LDA experiments, finding similar results across all experiments (Fig. 2e,f, gating strategy Supplementary Fig. 1B for WT). These findings demonstrate Tet− T cells are present in the naive T-cell repertoire at greater frequencies than Tet+ CD4+ T cells and proliferate in an antigen-specific manner that could be read out by the Nur77 assay.

### Lower-affinity T-cell clonotypes are identified by LDA

**Figure 3:**
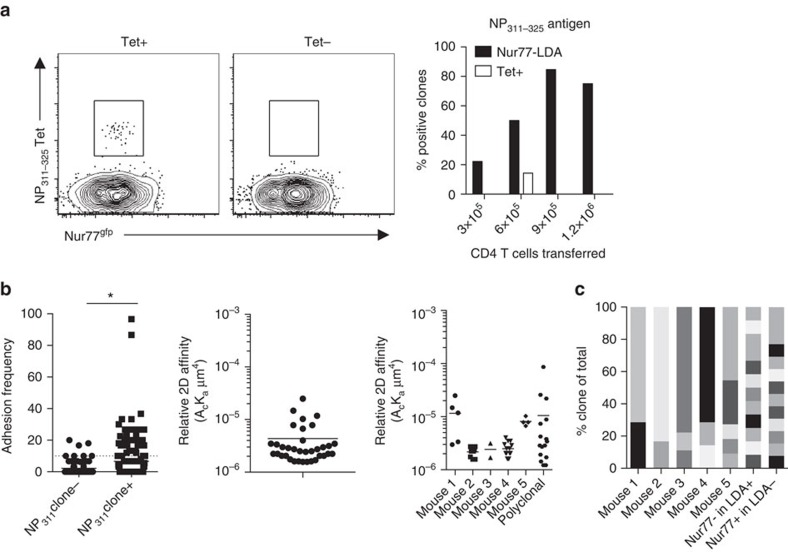
*In vivo* LDA identifies low-affinity CD4+ T-cell clones. (**a**) Representative NP_311–325_ Tet staining in TCRα^−/−^ LDA mice with both a Tet+ positive (left) and negative (right), with only 1 out of 34 mice possessing a Tet+-positive clone. (**b**) MP on single CD4+ T cells isolated from positive and negative LDA mice as identified by CD69/Nur77 (data combined from two independent experiments, *n*=2–3 mice, Student's *t*-test, *P=0.0073). MP affinities on individual positive LDA clones following NP_311–325_/CFA immunization along with a separate analysis of the polyclonal repertoire (single points represent individual cells). (**c**) Single-cell TCRβ sequencing from LDA-positive and -control mice. Individual colours represent unique TCRβ CDR3 sequences, with each column representing a single mouse.

To confirm the Nur77^gfp+^ CD4+ T cells identified Tet− T cells, pMHCII tetramer was used to costain unstimulated LDA samples when calculating precursor numbers (Fig. 3a). Of the 34 mice receiving T cells for calculating the precursor numbers for NP_311–325_, only one mouse possessed Tet+ T cells (Fig. 3a, left panel), while 20 mice possessed antigen-specific Nur77^gfp+^ cells and the remaining 13 mice did not respond. Next, the micropipette adhesion frequency assay (MP) was used to determine the affinity of CD4+ T cells from mice that were either positive or negative for antigen-specific Nur77 upregulation during LDA. CD4+ T cells from LDA-positive mice had a significantly greater adhesion frequency for the priming antigen NP_311–325_ (left panel, Fig. 3b), than mice with no measurable antigen-specific Nur77 upregulation, allowing for the calculation of TCR:pMHCII affinity (middle panel, Fig. 3b). The affinity for influenza NP_311–325_ was below that for which we previously reported was necessary for detection by MHC class II tetramers (>10^−4 ^μm^4^; right panel, Fig. 3b)^9^,^39^. When the affinity of T cells from five individual mice were assessed, mouse 2, 3, 4 and 5 displayed a range in affinity of <10-fold (Fig. 3b). TCR affinity ranges of 10-fold or less are characteristic of clonal T-cell populations^12^,^28^,^30^,^40^, while polyclonal populations can possess a 1,000-fold range in affinity^9^,^41^. Of note, mouse number 1 displayed a wider range of TCR affinity that appeared as distinct higher-affinity and lower-affinity populations, suggesting the presence of two clones. This is consistent with the frequency of T cells (1.2 × 10^6^ CD4+ T cells transferred) for that animal being above the limiting dilution level and the potential presence of more than one clone. A polyclonal assessment of TCR affinity for NP_311–325_ was included (Fig. 3b), displaying the wider affinity range (>100-fold) observed in polyclonal responses and demonstrating a similar range to the single clones measured (Fig. 3b). Overall, the micropipette analysis defined the presence of antigen-specific T cells with affinities below the minimum required for tetramer staining, while suggesting their clonality and confirming the antigen specificity of the functional Nur77^gfp^ LDA measurements.

To further demonstrate clonality of the LDA experiments, single-cell TCRβ sequencing was performed on Nur77^gfp^-positive and -negative populations from NP_311–325_ LDA mice. All LDA-positive mice were highly enriched for a single-TCRβ clonotype (>70%) with no TCR sequences shared between the mice (shared sequences identified as same colour in individual mice, Fig. 3c). The remaining sequences from each mouse correlated with the background green fluorescent protein expression identified in all Nur77^gfp^ animals. No TCRβ chain predominated in mice lacking antigen-specific T cells clones as defined by LDA or amongst the Nur77^gfp−^ T cells in a mouse with a positive clone identified by LDA (Fig. 3c). These data demonstrate the *in vivo* LDA with TCR repertoire analysis can identify and isolate single, lower-affinity T-cell clones and provides an effective method for calculating the precursor number of Tet− CD4+ T cells in the naive repertoire.

### T-cell expansion is correlative with naive T-cell numbers

**Figure 4:**
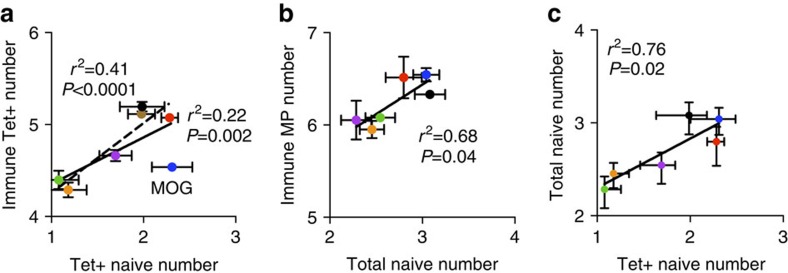
Total precursor numbers predict expansion of CD4+ T cells. Each point is a unique antigen-specific population: MOG_38–49_ (blue), GP_66–77_ (red), Aasf_24–32_ (purple), FliC_427–442_ (green), NP_311–325_ (orange), 85b_280–294_ (black), MOG_38–49_ from MOG KO (brown). (**a**) Number of naive Tet+ CD4+ T cells (log transformed) compared with Tet+ CD4+ T cells 14 days after immunization (log transformed; *n*=5–6 mice per group, data represented as mean±s.e.m.). (**b**) Number of antigen-specific CD4+ T cells (log transformed) identified by MP after immunization correlated with previously calculated precursor numbers (log transformed; expanded data: 12 experiments, two to three mice per point, data represented as mean±s.e.m.). (**c**) Correlation of the number of naive T cells (both log transformed) as identified by tetramer and LDA (data represented as mean±s.e.m.).

As we estimated the total antigen-specific CD4+ T cell for six epitopes in the naive mouse and found them to outnumber Tet+ counterparts, we wanted to next determine the contribution of the low-affinity CD4+ T cells on immune expansion. Naive Tet+ precursor frequency predicts the immunodominance of an antigen-specific T-cell population^4^,^8^, but these assays have not included Tet− CD4+ T cells or even those antigens enriched for lower-affinity TCRs such as self antigens like MOG. Analysis of foreign antigen-specific Tet+ CD4+ T cells after immunization with peptide in CFA confirmed the positive correlation between (*r*^2^=0.41, *P*<0.0001) precursor and expanded T-cell numbers (different antigens represented by each point, dotted line, Fig. 4a, gating strategy Supplementary Fig. 2). Yet, when MOG self-antigen-specific CD4+ T cells are included in the tetramer analysis (solid line, Fig. 4a) the *r*^2^ value decreases to 0.22 with a *P* value of 0.0021, indicating factors other than precursor frequency may contribute to Tet+ T-cell expansion to self-antigens^42^,^43^. Next, MP analysis of T cells from mice immunized 14 days earlier with peptide/CFA showed a strong correlation with the naive precursor frequency measured by LDA (Fig. 4b). When lower-affinity T cells measured by MP were included in the expanded T-cell numbers, the naive to expanded T-cell correlation improves, even with the inclusion of CD4+ T cells specific for MOG self-antigen (Fig. 4b). Further comparison of the precursor numbers from tetramer staining and LDA calculations revealed a significant correlation between the methods, suggesting tetramer can be used to roughly estimate the hierarchy within naive populations, though it still vastly underestimates naive T-cell numbers (Fig. 4c). In addition, MP identifies ∼10–150-fold greater numbers of expanded antigen-specific CD4+ T cells than by tetramer, significantly altering our understanding of the extent of CD4+ T-cell expansion.

**Figure 5:**
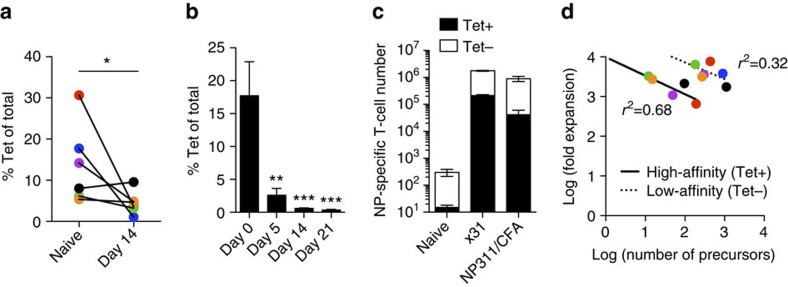
Low-affinity CD4+ T cells predominate CD4+ T-cell immune responses. Each point is a unique antigen-specific population: MOG_38–49_ (blue), GP_66–77_ (red), Aasf_24–32_ (purple), FliC_427–442_ (green), NP_311–325_ (orange), 85b_280–294_ (black) and MOG_38–49_ from MOG KO (brown). (**a**) Frequency of Tet+ CD4+ T cells in the naive and day 14 immunized repertoires (data shown as mean frequency for each antigen, paired Student's *t*-test, *P<0.05). (**b**) Frequency of MOG-Tet+ cells in the total MP+ T cells during the immune response to MOG_35–55_/CFA immunization (data shown as mean±s.e.m., one-way analysis of variance, **P<0.01, ***P<0.001 with day 0 as a reference value). (**c**) Enumeration of Tet+ and Tet− NP311-specific CD4+ T-cell contribution from either naive, day 10 post x31 influenza infection, or NP311/CFA immunizations (naive data taken from Fig. 4a, expanded data contain four independent experiments, with two mice each, data represented as mean±s.e.m.). (**d**) Plot of precursor number (log transformed) versus fold expansion (log transformed) of high-affinity and low-affinity T cells as identified by tetramer, LDA and MP.

To determine how TCR:pMHCII affinity influences the expansion of T cells during the primary immune response, the ratio of Tet+ to Tet− T cells were compared for all epitopes in both naive and immunized samples (Fig. 5a). No increase in the frequency of Tet+ T cells was found at the peak of expansion (day 14 after immunization), signifying Tet+ T cells did not gain a competitive advantage over lower-affinity T cells. In fact, a significant reduction in the frequency of Tet+ CD4+ T cells of the total expanding population was found for all antigens (Fig. 5a). This was not a function of the time point measured, as kinetic analysis of the MOG-specific repertoire revealed higher-affinity T cells contributed the most in naive state, with significantly less involvement as the immune response progressed (Fig. 5b). The large contribution of lower-affinity CD4+ T cells was also found in the NP_311–325_-specific T-cell population responding during influenza x31 infection and NP311/CFA immunization (Fig. 5c). This demonstrates that low-affinity T-cell recruitment does not only occur in response to CFA, but is equally present during infection. Next, the fold expansion of antigen-specific populations (Tet+ and Tet−, each point is a unique antigen) was graphed separately as a function of precursor frequency (Fig. 5d), finding CD4+ T-cell populations with smaller precursor numbers exhibiting greater expansion. MOG was removed from the analysis for higher-affinity T cells due to its altered expansion due to tolerance. When the higher-affinity (solid line) and lower-affinity (dotted line) populations were compared at the same precursor frequency, the lower-affinity T cells have the potential to expand to a greater number than the higher-affinity, Tet+ T cells (Fig. 5d). Interestingly, the slopes of the two lines generated are similar (Tet+: −0.47, Tet−: −0.42), signifying the two populations of cells compete within themselves comparably (Fig. 5d). Therefore, TCR:pMHCII affinity does not control the accumulation of CD4+ T cells during the immune response, and instead immune activation selects for a diverse range of affinities during primary immune expansion.

## Discussion

Precise quantification of T-cell precursor numbers and expansion is essential for understanding the function of the adaptive immune system, vaccine design and adoptive T-cell therapeutics. Initially, T-cell numbers were defined by *in vitro* LDA based on the frequency of functionally responsive cells^38^. TCR-Tg mice allowed for the study of the naive frequency and expansion of monoclonal populations, but did not address the diversity present in a polyclonal immune response^44^,^45^. The advent of pMHC tetramer technology began to address the limitation of monoclonal analysis by providing improved assessment of precursor and expanded T-cell numbers in more clonally diverse populations^4^,^7^. Key insight into the relationship between precursor numbers, expansion and cross-reactivity was provided with the use of the tetramers although tetramer-based affinity and avidity interactions do not fully encompass polyclonal T-cell responses, especially those enriched for lower-affinity interactions, that is ones specific for self-antigen^8^,^42^. Previous studies had identified these low-affinity, Tet− T cells, but have never developed a way to quantify, identify and phenotype these polyclonal T cells in their naive or activated state^11^,^25^. Therefore, this work adds to these initial observations, allowing for the study of low-affinity, Tet− T cells in a polyclonal model, providing increased depth of understanding to CD4+ T-cell responses.

A major goal of this work was to quantify the precursor number of antigen-specific CD4+ T cells inclusive of lower-affinity T cells missed by MHC class II tetramers. In calculating the total naive T-cell numbers, we chose to perform these experiments in T-cell-deficient mice. As the LDA is a digital response (cells are either present or absent), the lymphopenic environment increases the signal to noise ratio of the assay by allowing for larger proliferation of the single clone being measured for Nur77 expression after restimulation. The lymphopenic environment has minimal impact on the competition dynamics between high-affinity and low-affinity T cells, as LDA calculations were similar between mice with (WT) and without (TCRα−/−) T cells. Therefore, we can conclude the lymphopenic environment has minimal impact on competition dynamics between high-affinity and low-affinity T cells in the LDA calculations. This is in agreement with previous work as groups have suggested the initial precursor frequency of CD4+ T cells is low enough to prevent the competition between antigen-specific T cells^24^. Once T cells have expanded, some infer that competition for resources could favour the dominance of individual clonotypes that many would presume relate to TCR affinity^19^,^20^. Instead for all polyclonal responses analysed here, we find a distribution of TCRs where low-affinity CD4+ T cells expand from their naive numbers to remain more numerous in the immune repertoire. On secondary challenge, both high-affinity and low-affinity T cells have been shown to have an advantage in survival^19^,^22^. We hypothesize that there will be narrowing of the antigen-specific TCRβ population, as only some of the clones will respond to antigen, but between the high-affinity and low-affinity populations there will be no enhanced survival. This is likely due to mechanisms that can modulate TCR signalling such as TCRβ downregulation^22^ and Lck-coreceptor conjugation^46^, which have been shown to occur after primary immune responses.

Our data indicate TCR affinity does not predict the peak expansion of T cells in response to primary antigen exposure, though we do not know if affinity affects the efficiency of entry into the immune response. The correlation between affinity and expansion has been proposed before, but conflicting data exists. For example one could conclude that affinity does not correlate with expansion to antigen based on Tg-barcoding experiments^47^,^48^. In these experiments, a single OT-I T cell can have a range of contribution to the expanded repertoire even though each T-cell expressed the same clonal TCR^47^,^48^. As well, high-affinity and low-affinity CD4+ T cells show similar efficiency of proliferation in both *in vitro* and *in vivo* work^22^,^28^. On the other hand, the use of altered peptide ligands (APLs) or a fixed TCRβ chain Tg has demonstrated the magnitude of expansion and contribution to the total repertoire was correlative with TCR:pMHC affinity^21^,^23^. It is unclear what factors are different between these experiments, but potentially infection type, TCR-Tg T-cell thymocyte development or competition with the endogenous repertoires may affect competition and expansion. Thymocyte development has been shown to play an important role in setting the basal activity of T cells^49^, but TCR-Tg T cells would not undergo these varied developmental consequences, thereby potentially altering an important negative regulatory loop in T-cell development with different affinities. Our data based on the polyclonal T-cell response to six different antigens indicates that TCR affinity does not influence clonal expansion dominance.

The identification of low-affinity CD4+ T cells always comes with questions about the functionality of this T-cell subpopulation, as it is hypothesized that low-affinity equates to sub-optimal and that the enumeration of Tet+ and functional responses leads to similar magnitudes^6^,^50^. However, these assumptions are not completely accurate. Transcription factor profiling of the CFA immune response has shown Tet+ cells have at most 20% T-bet+ (T_H_1 lineage-defining transcription factor) expression^8^,^43^. In contrast, experiments monitoring cytokine secretion by T cells in this same immune response have show interferon-γ (IFN-γ) enzyme-linked immunoSpot (ELISPOT) data and Tet+ T-cell number equate^8^,^42^. Therefore, Tet+ T-bet+ CD4+ T cells cannot be the sole source of IFN-γ production in ELISPOT experiments. Likely, lower-affinity T cells are contributing to this pool of antigen-specific T cells identified by ELISPOT. Recent work using a pMHCII dodecamer (12 pMHCII arms instead of four) supports this hypothesis as they found Tet−, but dodecamer+ T cells exhibited similar function to Tet+ T cells^51^. The dodecamer reagent, while giving increased numbers as compared with tetramers, only showed two to three times greater identification of T cells, which is still an underestimation as compared with the seven to eight times increase we find using Nur77 in the naive repertoire and >10 × increases found using the micropipette. Please note, that Nur77, like all functional responses, underestimates the numbers of CD4+ T cells in an immune response as not every T cell can respond at a given time. For example, analysis of cloned TCRs in a retrogenic system found that several retrogenic-TCRs (TCR-Rg) could cause autoimmune diabetes^30^, but within each TCR-Rg group, only a fraction could upregulate Nur77 even though the population shared the same TCR. Therefore, induction of Nur77 expression as readout by the reporter is likely less sensitive then the measurement of effector functionality and likely independent of TCR affinity. More work will be needed to understand the interaction of TCR affinity, T-cell signalling thresholds and their correlation with effector function.

Since polyclonal TCR affinities during the CD4+ T-cell response are maintained from the naive state, this diversity most likely serves a functional purpose, as biological systems are seldom wasteful. In T-cell immunotherapeutics, some TCRs have been engineered for higher-affinity pMHC interactions with the belief that the highest-affinity TCR would generate the most efficacious immunodominant response^52^. Of interest, the selection of engineered higher-affinity TCRs has been both successful and disastrous in patients with outcomes that have included death^53^,^54^. This points to a need for further understanding of what is an optimal affinity range for effective immunity, with recent data showing greater function of TCRs with intermediate affinity^22^. Instead of a single unusually high-affinity TCR, a range of affinities might prove more advantageous. Our data demonstrates that population diversity is a property of the immune response and that mechanisms maintain a diverse affinity range of CD4+ T cells in a polyclonal population. For example, population diversity of antigen responsive T cells can be seen in the production and use of interleukin-2. While only a subset of T cells produce interleukin-2, both high-affinity and low-affinity T cells may use this key growth cytokine^36^,^55^,^56^. A counterpoint to the concept of favoring higher-affinity T cells are the findings that lower-affinity T cells possess preferred differentiation patterns, with these T cells more likely to acquire T_H_2, T_FH_ or T_CM_ phenotypes^6^,^36^,^50^,^57^,^58^. As well, data has shown that initial induction of peripherally derived regulatory T cells can arise from lower-affinity TCR:pMHCII interactions^59^,^60^. By understanding the population characteristics of lower-and higher-affinity CD4+ T cells together, unique immune treatments may be developed with targeted characteristics.

Inclusion of lower-affinity TCRs in immune repertoires leads to a large increase in the numbers of CD4+ T cells specific for a single epitope, altering our understanding of TCR cross-reactivity. TCR cross-reactivity, defined as a single αβ TCR binding to multiple pMHC, has been shown to be necessary for complete immune protection against pathogens, as there are a greater number of potential epitopes (20^9^∼5.12 × 10^11^) than estimated mouse αβ TCR clonotypes (2 × 10^6^)^2^,^61^. Our findings of increased CD4+ T cells specific for a single antigen increases the theoretical amount of TCR cross-reactivity required for complete immune protection by eightfold^2^. Due to the increase in cross-reactivity, the amount of T cells needed to protect an entire mouse, termed the protecton, may be similarly decreased due to the increased number of T cells for a single antigen^3^. Future work will need to clarify how affinity impacts cross-reactivity or if there is any correlation at all.

In conclusion, we find the expansion of naive CD4+ T cells in the primary immune response is independent of TCR:pMHCII affinity, while also quantitating the total number of CD4+ T cells in an immune response. Quantitation of the total repertoire reveals that up to 90% of the CD4+ T cells participating in the immune response are ignored by conventional analyses. It will be of interest to determine if this frequency of ignored CD4 T cells is a constant or if it can be altered based on the antigen delivery. Potentially, priming antigen doses used here could affect the ratio of high-affinity to low-affinity T cells, as it has been shown antigen dose changes the expansion and differentiation of high-affinity, Tet+ CD4+ T cells^26^. However, recent work has shown use of low-antigen concentration to activate CD4+ TCR-Tg T cells with different affinities for the same antigen causes similar primary division rates, thereby maintaining the diversity of the T-cell population even across a range of affinities^22^.Since lower-affinity CD4+ T cells have been shown to have similar roles as higher-affinity T cells^26^,^36^, the sole use of pMHCII tetramers underestimate the diversity and richness of the immune system by not monitoring these lower-affinity cells. The expansion and continual presence of these T cells likely highlight the need of affinity diversity for maintenance of a healthy immune system and limiting microbial immune evasion^62^,^63^,^64^. Future studies are needed to fully understand how low-affinity T cells may impact human immune health, as we predict to see similar total numbers of antigen-specific T cells in mice and humans given that they possess similar repertoire diversity and specificity^2^,^65^.

## Methods

### Mice

C57BL/6NCr (WT) mice were purchased from the National Cancer Institute, while MOG KO mice^66^ were a gift from Hugh Reid and were bred on site. Thy1.1+, Nur77^gfp^ and TCRα−/− mice were purchased from Jackson Laboratories and were bred on site. Mice were 6–8 weeks old when used for experiments. Both males and females were used. WT mice immunized with MOG_35–55_ were monitored for weight loss due to experimental autoimmune encephalomyelitis (EAE) and were killed if weights fell <20% of initial starting weight. Experimental sample sizes were chosen from previous experiments on naive and expanded T-cell numbers^8^. No mice were excluded from analysis. No randomization was performed for experiments and no investigator blinding was performed. All animals were housed in an Emory University Department of Animal Resources facility (Atlanta, GA, USA). Permission was granted and performed in accordance with the protocols of the Institutional Animal Care and Use Committee.

### Peptide priming

MOG_35–55_ (MEVGWYRSPFSRVVHLYRNGK), 85b_280–294_ (FQDAYNAAGGHNAVF) GP_61–81_ (GLKGPDIYKGVYQFKSVEFD), Aasf_24–32_ (VSSPAVQES), NP_311–325_ (QVYSLIRPNENPAHK) and FliC_427–441_ (VQNRFNSAITNLGNT) peptides were synthesized on a Prelude peptide synthesizer (Protein Technologies, Inc., Tuscon, AZ, USA). For all peptide immunizations, 200 μg of the peptide was emulsified in 375 μg of CFA and injected subcutaneously into the flank of a mouse on days 0 and 7 (150 μl total volume per injection). CFA was made in-house by mixing 20 ml of Incomplete Freund's Adjuvant (Becton Dickinson, Franklin Lakes, NJ, USA) and 100 mg of desiccated *Mycobacterium tuberculosis* H37 Ra (Becton Dickinson). On days 0 and 2, 300 ng of pertussis toxin (Ptx, List Biological Labratories, Campbell CA, USA) was injected intraperionteallly in all immunizations to compare both the self and foreign immune responses.

### Tetramer enrichments

Tetramers and monomers were provided by the National Institute of Allergy and Infectious Diseases Tetramer Core Facility at Emory University or were a generous gift of Marc Jenkins. Tetramer enrichment and staining was performed as previously described^67^. Briefly, mouse peripheral lymphoid organs (spleen and inguinal, para-aortic, brachial, axillary, cervical and mesenteric lymph nodes) were processed into a single-cell suspension. Cells were then stained with the respective tetramer (phycoerythrin (PE)- and/or allophycocyanin (APC)-conjugated, 4 μg ml^−1^ final concentration) for 60 min at room temperature, washed, stained with 50 μl of anti-PE or anti-APC magnetic microbeads for 30 min on ice (Miltenyi Biotec, Germany), washed and enriched on a magnetized LS column (Miltenyi Biotec). The bound and flow-through samples were then sampled to determine population counts using AccuCheck microbeads (Invitrogen, Carlsbad, CA, USA) and stained for analysis by flow cytometry. Antibodies used are show in Supplementary Table 1. For intracellular staining, cells were treated with the Tonbo or eBioscience Fixation and Permiabilization kits as per the manufacturer protocol. Samples were collected on an LSR II (Becton Dickinson) and analysed using FlowJo (Treestar, Ashland, OR, USA).

### CD4+ T-cell adoptive transfer

Splenocytes from naive mice were collected and processed into a single-cell suspension. CD4+ T cells were purified following manufacturer instructions using the CD4+ T-cell negative isolation kit (Miltneyi Biotec). Purified CD4+ T cells were analysed by flow cytometry for purity and counted by flow cytometry using AccuCheck microbeads (Invitrogen). Purified CD4s were injected intravenously into recipient mice and immunized 24 h later. Park rate at 24 h was measured in TCRα^−/−^ and found to be ∼20% (Supplementary Fig. 3).

### Nur77 analysis

For experiments comparing donor high-affinity and low-affinity T cells in a WT mouse using tetramers, spleens and lymph nodes from recipient, immunized mice were collected and processed into single-cell suspensions. Tetramer enrichment was performed as described above. Bound samples were then analysed by flow cytometry. For adoptive transfer limiting dilution experiments in WT mice, Thy1.2 enrichment was performed using anti-Thy1.2 antibody and anti-APC magnetic Microbeads (Miltenyi Biotec) following manufacturer protocol. For Nur77 analysis in WT mice flow-through (FT) samples were used and not enriched. For all cases, prepared samples were stimulated for 18–22 h in quadruplicate with 10 μg ml^−1^ of peptide (one specific peptide and three non-specific peptides). Samples were then collected and stained for analysis of donor (Thy1.2+) CD4+ T-cell Nur77 upregulation by flow cytometry. For analysis of the frequency of Nur77 upregulation, non-specific background was averaged and subtracted from both specific and non-specific samples, and then graphed. Discrimination of positive and negative clones for LDA was performed as described in the section on calculations with the values being reported as mean±95% confidence intervals.

For experiments calculating naive precursor frequency of low-affinity CD4+ T cells, spleens from recipient TCRα^−/−^ mice were collected and processed individually into single-cell suspensions. Splenocytes (2–3 × 10^6^) from each mouse were plated in quadruplicate, with three samples stimulated with 100 μg ml^−1^ of peptide for 18–22 h and one sample remaining unstimulated. The unstimulated sample was used for pMHCII tetramer staining to detect higher-affinity CD4+ T cells. Stimulated splenocytes were collected, stained with antibodies shown and analysed by flow cytometry as described above. Discrimination of positive and negative clones was performed as described in the section on calculations.

### Nur77 functional measurement

Spleen and lymph nodes of previously immunized mice were processed into a single-cell suspension and counted. Samples were split in half and both set of cells were stimulated with 10 μg ml^−1^ of peptide at a concentration of 1 × 10^7^ cells per ml in complete media (RPMI 1640, 10% (v/v) FCS, 2 mM L-glutatmine, 0.05 mM 2-mercaptoethanol and 0.05 mg ml^−1^ gentamicin sulfate) for 4 h. One sample received MOG_35–55_ (antigen specific), while the other received GP_61–81_ (non-specific). Samples were then collected and tetramer enrichment was performed as described above. Both bound and flow-through samples were then analysed by flow cytometry.

### TCRβ sequencing

Single-cell *Tcrb VDJ* sequencing was performed as previously described^68^. In preparation for sequencing, LDA experiments were performed for the NP_311–325_ antigen in TCRα^−/−^ mice (see section on LDA). After restimulation and flow cytometry, the samples were analysed to determine if they possessed a low-affinity T-cell clone (see section on Calculations). Single CD4+ T cells from positive and negative LDA samples were then index-sorted by a FACS Aria II (Becton Dickinson) into a 96-well plate containing 2.5 μl cDNA master mix (iScript cDNA Synthesis Kit, Bio-Rad). Column 12 of the 96-well plate did not receive cells, thereby acting as a negative control wells for each plate. After production of complementary DNA, nested *Tcrb VDJ* PCRs were performed on each sample and the negative control column was confirmed by gel electrophoresis. Samples were then sent to Beckman Coulter Genomics (Danvers, MA, USA) for Sanger sequencing. Individual sequences were tabulated and parsed by in-house designed software and then analysed by The International Immunogenetics Information system (IMGT)^69^,^70^,^71^. Non-productive sequences were not analysed.

### TCR affinity measurement

Spleens from immunized mice were removed on the noted days and processed into a single-cell suspension. CD4+ T cells were purified using the CD4+ T-cell positive selection kit (Miltenyi Biotec) as per manufacturer instructions. In parallel, CD4+ T cells were counted by flow cytomtery using AccuCheck beads as described above. Red blood cells (RBCs) were isolated in accordance with the Institutional Review Board at Emory University and prepared as previously described^12^. RBCs coated with various concentrations of Biotin-X-NHS (EMD) were coated with 0.5 mg ml^−1^ streptavidin (Thermo Fisher Scientific, Waltham, MA, USA), followed by 1–2 μg of pMHCII monomer. The pMHCII-coated RBCs were stained with anti-MHC class II PE antibody, and purified T cells were stained with anti-TCRβ (eBioscience, H57-597) PE antibody. The densities of I-A^b^ and TCR were calculated using BD QuantiBrite Beads (Becton Dickinson). The micropipette adhesion frequency assay was then preformed as previously described^12^. In brief, a pMHC-coated RBC and T cells were placed on opposing micropipettes and brought into contact by micromanipulation for a controlled contact area (A_c_) and time (t). The T cell was retracted at the end of the contact period, and the presence of adhesion (indicating TCR–pMHC binding) was observed by elongation of the RBC membrane. This TCR–RBC contact was repeated 25 times and the adhesion frequency (Pa) was calculated. The relative 2D affinity (A_c_K_a_) of each cell that had a Pa of >10% was calculated using the Pa at equilibrium (where t→∞) using the following equation: A_c_K_a_=−ln[1−Pa(∞)]/(mrml), where mr and ml reflect the receptor (TCR) and ligand (pMHC) densities, respectively. The total frequency of cells that bound to pMHCII-coated RBCs was tabulated and used for the calculation of antigen-specific CD4+ T-cell numbers below. Previous reports have shown that as few as 10 cells in a polyclonal population need to be ran to generate an average affinity for the population, while considerably fewer cells (estimated to be five to seven cells) in a monoclonal repertoire need to be measured for an average affinity^6^. For each antigen, the number of binders and cells ran is as follows (shown as binders/cells ran): WT MOG_35–55_ (33/95), KO MOG_35–55_ (27/80), GP_61–81_ (38/174), FliC_427–441_ (11/154), Aasf_24–32_ (11/165), NP_311–325_ (17/249) and 85b_280–294_ (32/208).

### Influenza x31 infections

WT mice were infected intranasally with influenza A/HKx31 (H3N2) at 30,000 EID_50_ (50% egg infectious doses) as previously described^72^. Spleens were collected at day 10 post infection. Magnetic enrichment was performed using CD4+-positive selection following manufacturer protocol (Miltenyi Biotec). Purified cells were then stained with 4 μg ml^−1^ NP311:I-A^b^ PE tetramer for 60 min at room temperature or used in the MP assay to determine the number of antigen-specific cells.

### Calculations

For Nur77^gfp^ LDA experiments, all samples were stimulated with their immunized antigen (100 μg ml^−1^) and two to three other non-specific antigens (100 μg ml^−1^). The frequencies of CD44^+^Nur77^gfp+^CD69^+^ CD4+ T cells were tabulated from specific and non-specific antigen controls. Samples were determined to be positive if the frequency of Nur77^gfp+^CD69^+^ CD4+ T cells in the antigen-specific sample was three s.d.'s above the mean of the averaged non-specific controls. The number of antigen-specific T cells from the LDA curves was calculated using an online calculator from a previously described method and reported as mean ± the 95% confidence interval^73^.

To calculate the number of CD4+ T cells specific for a given antigen by MP, the frequency of non-specific binders was determined by performing the MP assay on CD4+ T cells from mice immunized with non-specific peptide in CFA (Supplementary Fig. 4). These background-binding frequencies were subtracted from the frequencies generated in antigen-specific experiments and total numbers of antigen-specific CD4+ T cells were calculated from previously generated absolute counts of CD4+ T cells in the spleen.

### Statistical analysis

One-way analysis of variance, two-tailed, unpaired Student's *t*-tests, linear regression and two-tailed Student's *t*-tests were performed using Prism (GraphPad, LaJolla, CA, USA) Software.

### Data availability

The data and analysis software that support the findings of this study are available from the corresponding author on request.

## Additional information

**How to cite this article:** Martinez, R. J. *et al*. Low-affinity CD4+ T cells are major responders in the primary immune response. *Nat. Commun.* **7,** 13848 doi: 10.1038/ncomms13848 (2016).

**Publisher's note:** Springer Nature remains neutral with regard to jurisdictional claims in published maps and institutional affiliations.

## Supplementary Material

Supplementary Information Supplementary Figures 1-4, Supplementary Table 1 and Supplementary References.
