# Supplementary material for: Low-affinity CD4+ T cells are major responders in the primary immune response
Source: Nat Commun. 2016 Dec 15;7:13848. doi: 10.1038/ncomms13848 (PMC5234832; doi:10.1038/ncomms13848)
Supplement: Supplementary Information — Supplementary Figures 1-4, Supplementary Table 1 and Supplementary References. [file ncomms13848-s1.pdf]

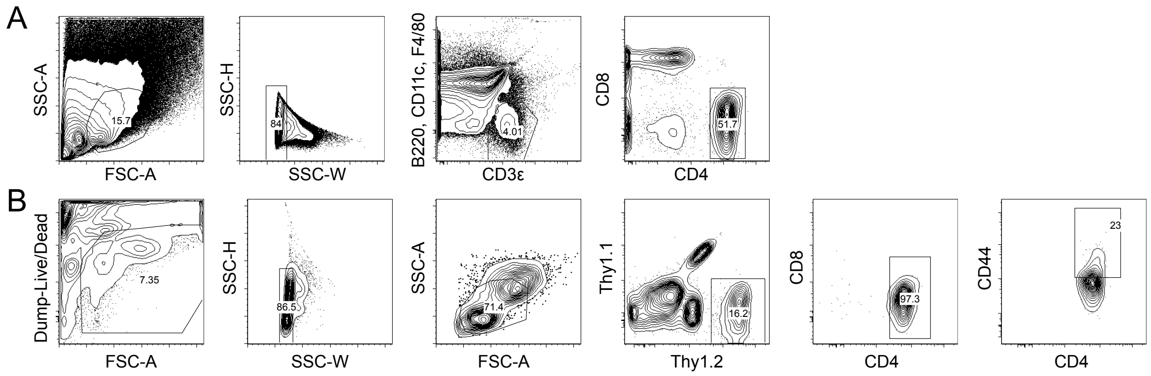

Supplementary Figure 1. Identification of antigen-specific donor cells in WT LDA. (A) Gating strategy for Thy1.2 CD4 T cell transfer into WT (Thy1.1) mice following tetramer+ LDA. (B) Gating strategy for Thy1.2 CD4 T cells into Thy1.1 mice for LDA low-affinity CD4 T cell calculations.

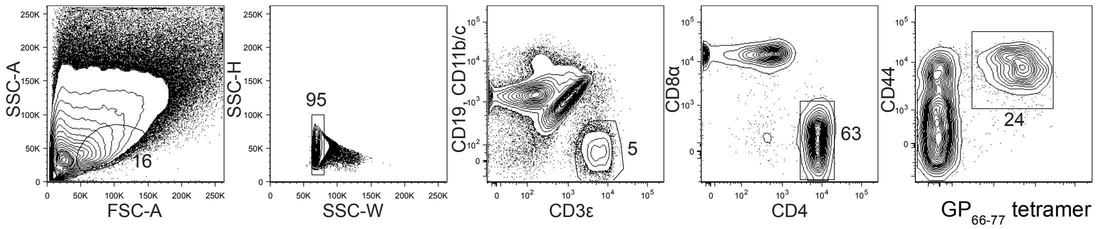

Supplementary Figure 2. Identification of antigen-specific CD4+ T cells by tetramer.

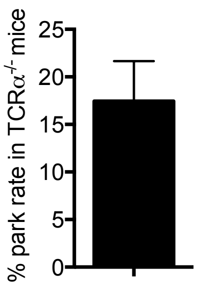

Supplementary Figure 3. Park rate of CD4 T cells 24 hours after IV transfer into $TCR\alpha^{-/-}$ mice as a percent of input (n=4 mice, two experiments, data shown as mean +/- SEM).

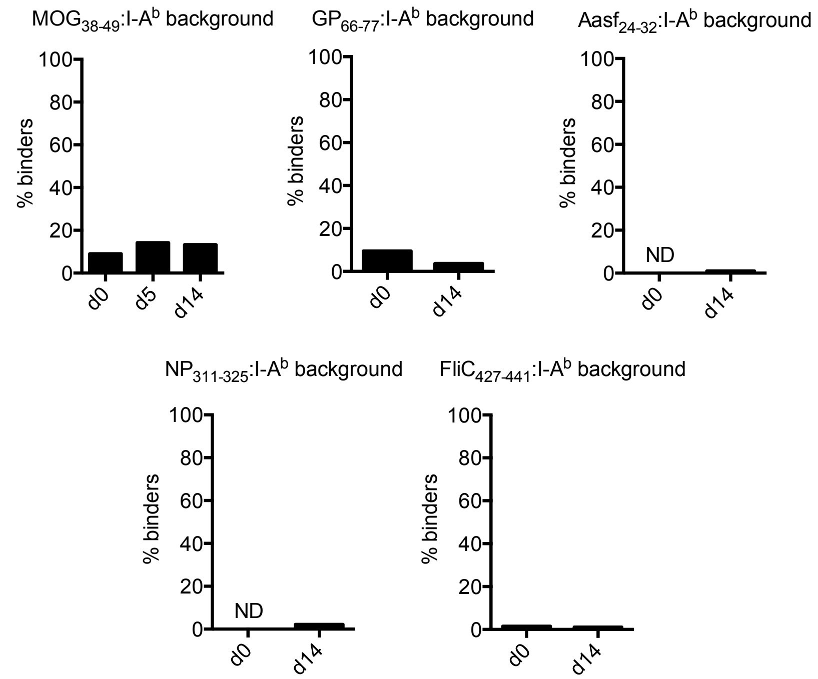

Supplementary Figure 4. MP background calculation (data shown as mean frequency of binders for all points, 2 mice per timepoint).

Target	Fluorochrome	Clone	Host	Isotype	Manufacturer	Cat#	Reference
CD3e	BV 605	145-2C11	Hamster	IgG1,κ	Biolegend	100351	1
CD4	BV 510	RM4-5	Rat	IgG_{2a}, κ	Biolegend	100553	2
CD8α	V450	53-6.7	Rat	IgG_{2a}, κ	Tonbo Biosciences	75-0081-U100	3
CD11b	PerCp/APC Cy7	M1/70	Rat	IgG_{2b}, κ	BD Pharmigen	561114 / 561039	4
CD11c	PerCp/ APC-Cy7	HL3	Hamster	IgG1, λ2	BD Pharmigen	560584 / 561241	5
CD19	PerCp	1D3	Rat	IgG_{2a}, κ	BD Pharmigen	551001	6
CD44	AF700	IM7	Rat	IgG_{2b}, κ	eBioscience	56-0441-82	7
CD69	BV605	H1.2F3	Hamster	IgG1, λ3	BioLegend	104529	8
F4/80	APC-Cy7	BM8	Rat	$IgG_{2a,}$ κ	eBioscience	47-4801	9
Nur77	PE	12.14	Mouse	IgG1	eBioscience	12-5965	10
TCRβ	PE	H57-597	Armenian Hamster	IgG	eBioscience	12-5961	11
B220	APC-efluor780	RA3-6B2	Rat	IgG_{2a}, κ	eBioscience	47-0452	12
Thy1.1	PerCp	HIS51	Mouse	$IgG_{2a,}$ κ	eBioscience	45-0900	13
Thy1.2	APC	53-2.1	Rat LOU	IgG_{2a}, κ	BD Pharmigen	561974	14
I-A/I-E	PE	M5/114.15.2	Rat BNxLEW	$IgG_{2b,}$ κ	BD Pharmigen	557000	15
Bcl-6	PE-CF594	K112-91	Mouse	$IgG_{1,}$ κ	BD Horizon	562401	16

Supplementary Table 1. Antibodies used in experiments.

References

1. Leo O, Foo M, Sachs DH, Samelson LE, Bluestone JA. Identification of a monoclonal antibody specific for a murine T3 polypeptide.Proc Natl Acad Sci U S A. 1987 Mar;84(5):1374-8
2. Shi Y, Kaliyaperumal A, Lu L, Southwood S, Sette A, Michaels MA, Datta SK. Promiscuous presentation and recognition of nucleosomal autoepitopes in lupus: role of autoimmune T cell receptor alpha chain. J Exp Med. 1998 Feb 2;187(3):367-78.
3. Ledbetter, J. A. and L. A. Herzenberg. Xenogeneic monoclonal antibodies to mouse lymphoid differentiation antigens. Immunol Rev. 1979;47:63-90.
4. Springer, T., G. Galfre, et al. 1978. Monoclonal xenogeneic antibodies to murine cell surface antigens: identification of novel leukocyte differentiation antigens. Eur J Immunol 8(8): 539-51. / Springer, T., G. Galfre, et al. 1979. Mac-1: a macrophage differentiation antigen identified by monoclonal antibody. Eur J Immunol 9(4): 301-6.
5. Metlay JP, Witmer-Pack MD, Agger R, Crowley MT, Lawless D, Steinman RM. The distinct leukocyte integrins of mouse spleen dendritic cells as identified with new hamster monoclonal antibodies. J Exp Med. 1990; 171(5):1753-

1771 / Pulendran B, Lingappa J, Kennedy MK, et al. Developmental pathways of dendritic cells in vivo: distinct function, phenotype, and localization of dendritic cell subsets in FLT3 ligand-treated mice. J Immunol. 1997; 159(5):2222-2231

6. Krop I, Shaffer AL, Fearon DT, Schlissel MS. The signaling activity of murine CD19 is regulated during cell development.J Immunol. 1996 Jul 1;157(1):48-56.
7. Trowbridge, I. S., J. Lesley, et al. 1982.Biochemical characterization and cellular distribution of a polymorphic, murine cell-surface glycoprotein expressed on lymphoid tissues. Immunogenetics 15(3): 299-312.
8. Yokoyama, W. M., F. Koning, et al. (1988). "Characterization of a cell surface-expressed disulfide-linked dimer involved in murine T cell activation." J Immunol 141(2): 369-76.
9. Leenen PJ, de Bruijn MF, Voerman JS, Campbell PA, van Ewijk W. Markers of mouse macrophage development detected by monoclonal antibodies. J Immunol Methods. 1994. 174(1-2):5-19.
10. Hazel TG, Mirsa R, Davis IJ, Greenberg ME, Lau LF. Nur77 is differently modified in PC12 cells upon membrane depolarization and growth factor treatment. Mol Cell Biol. 1991; 11(6):3229-3246.
11. Kubo RT, Born W, Kappler JW, Marrack P, Pigeon M. Characterization of a monoclonal antibody which detects all murine alpha beta T cell receptors. J Immunol. 1989 Apr 15;142(8):2736-42.
12. Morse, H. C. d., W. F. Davidson, et al. 1982. Abnormalities induced by the mutant gene Ipr: expansion of a unique lymphocyte subset. J Immunol. 129(6): 2612-5.
13. Hermans, M. H. and D. Opstelten 1991. In situ visualization of hemopoietic cell subsets and stromal elements in rat and mouse bone marrow by immunostaining of frozen sections. J Histochem Cytochem 39(12): 1627-34.
14. Ledbetter, J. A. and L. A. Herzenberg. Xenogeneic monoclonal antibodies to mouse lymphoid differentiation antigens. Immunol Rev. 1979;47:63-90.
15. Bhattacharya A, Dorf, M E and Springer T A. A shared alloantigenic determinant on Ia antigens encoded by the I-A and I-E subregions: evidence for I region gene duplication. J Immunol 1981 127:2488-95.
16. Choi, Kageyama, Eto et al.: "ICOS receptor instructs T follicular helper cell versus effector cell differentiation via induction of the transcriptional repressor Bcl6." in: Immunity, Vol. 34, Issue 6, pp. 932-46, 2011
